# In situ genetic engineering of host T-cells based on acellular scaffold strategy: a big but also small step for solid tumor immunotherapy

**DOI:** 10.1186/s40779-024-00517-8

**Published:** 2024-02-03

**Authors:** Shu-Yan Han, Zi-Xuan Zhao, Jun Wu

**Affiliations:** 1 Bioscience and Biomedical Engineering Thrust, the Hong Kong University of Science and Technology (Guangzhou), Nansha, Guangzhou, 511400 Guangdong China; 2 Department of Nephrology, Center of Kidney and Urology, the Seventh Affiliated Hospital of Sun Yat-Sen University, Shenzhen, 518107 China; 3 Division of Life Science, the Hong Kong University of Science and Technology, Hong Kong, 999077 China

**Keywords:** Acellular biomaterial scaffold, T-cell therapy, Solid tumor immunotherapy, Biomaterial-biosystem interaction, Multidisciplinary and interdisciplinary, Clinical transformation

The advent of targeted T-cell therapy, with chimeric antigen receptor (CAR) T-cell therapy as the most prominent example, has yielded significant clinical efficacy for both relapsed and refractory hematological malignancies. However, this form of T-cell immunotherapy is often accompanied by severe systemic toxicities, suboptimal response rates, and host immune rejection in clinical settings, which detracts from its therapeutic utility. Additional concerns, such as the time-intensive ex vivo manufacturing process and the substantial treatment costs, also require resolution. Beyond these limitations, the use of CAR T-cell therapy against solid tumors presents an ongoing and formidable challenge. The extensive heterogeneity and complex spatial organization of solid tumors, along with their associated microenvironments, have impeded the broader clinical adoption of T-cell-based tumor immunotherapies [1, 2].

In the work of Dandia et al. [3], a novel strategy was reported that utilizes an acellular three-dimensional scaffold-based localized approach to program host T cells in situ, thus addressing several major challenges faced by traditional T-cell therapies and offering new hope for the elimination of solid tumors. The polyethylene glycol (PEG) scaffolds, conjugated with poly-L-lysine (PLL) and loaded with ovalbumin (OVA)-specific T-cell receptors (TCRs) lentiviruses (LVs), were implanted in B16-OVA melanoma-bearing mice and demonstrated significant anti-solid tumor efficacy. These bioactive scaffolds effectively recruited host T cells to the tumor site, transduced them with OVA-specific TCRs, and enabled them to home to tumors and draining lymph nodes. This facilitated in vivo T-cell genetic engineering and solid tumor immunotherapy. On one hand, this approach circumvented the need for in vitro manipulation and large-scale expansion of allogeneic T cells by directly utilizing host cells, thereby reducing the common risks associated with traditional adoptive cell therapies. On the other hand, unlike systemic delivery, the scaffold-based in situ localized administration minimized the incidence of “on-target, off-tumor” effects and enhanced the efficiency of regional immunomodulation, making it particularly effective at overcoming immunosuppression within solid tumors.

As is widely recognized, the ultimate goal of preclinical research is to facilitate successful clinical translation into practical medicine. The unquestionable benefits of this novel in situ immunomodulation strategy include its streamlined one-step, one-day process, as well as its high-efficiency targeting and programming of solid tumors, which engender considerable optimism for the immunotherapy of solid tumors with its ease of operation, reduced cost, and exceptional efficacy. However, the promising results of the current proof-of-concept study represent merely the beginning, and numerous considerations must be addressed before this approach can be applied clinically.

First of all, biosafety remains the cornerstone of engineered cell therapy design. In the context of lentiviral delivery, the incorporation of foreign viral envelope antigens and the genomic integration of substantial DNA segments adjacent to active genes may heighten the risk of adverse effects. Moreover, these procedures may elicit unforeseen innate immune reactions that could compromise the stability and targeted specificity of the viral vectors. Consequently, it is imperative that the forthcoming generation of lentiviruses is both safe and efficacious for clinical applications. Furthermore, while the bioactive scaffold strategy represents a viable solution that could economize time, financial resources, and effort after treatment, the challenge of mass-producing a sufficient quantity of potent and active virions prior to product commercialization remains formidable [1, 4].

Secondly, the role of biomaterials as integral components of in situ therapeutic strategies and pivotal elements in market transition necessitates a deeper exploration of the structure–property relationships and the potential immunomodulatory mechanisms inherent to the scaffolds. The study at hand concludes that negatively charged LVs can be anchored to matrices through PLL modification, allowing host immunocytes to infiltrate the implants without complete immobilization [3]. Indeed, PLL-based electrostatic interactions are not confined to material-material and material-cell interfaces but may extend directly to material-receptor interactions. Nevertheless, a paucity of detailed mechanistic studies exists, which hampers the full understanding of the influence of polymeric scaffolds on diverse immune cells. In addition, the field has recently seen the emergence of novel self-therapeutic biomaterials for tumor treatment; these include synthetic amphiphilic cationic polymers that emulate oncolytic peptides (termed oncolytic polymers) [2] and hydroxy acid homopolymers derived from Chinese herbs. Owing to their advantageous chemical processability, cost-effectiveness, abundant availability, and scalability, these functionalized biomaterials stand out as promising contenders for a new therapeutic paradigm. Their synergistic anti-tumor properties have the potential to refine the clinical application of pure cell therapies and to complement existing cancer immunotherapy approaches.

Lastly, the challenges posed by tumor antigen escape and heterogeneity, particularly in solid tumors, render the elucidation of genetic and epigenetic diversity and the underlying operational mechanisms essential for the tailored treatment of individual tumors and the standardized production of therapeutic agents. Undoubtedly, there is an urgent need for increased interdisciplinary collaboration and the integration of novel techniques [5, 6]. For instance, the molecular signaling pathways and potential mechanisms governing immune cell activities can be elucidated with high-resolution, spatiotemporally controlled single-cell imaging techniques; personalized immunotherapy can benefit from neoantigen discovery and screening, facilitated by high-throughput sequencing, machine learning, and computational prediction. Additionally, the integration of multiple spatial omics, artificial intelligence, and clinical oncology is instrumental in constructing interactive networks within the tumor microenvironment and enhancing clinical management of patients. As technological advancements continue at a swift pace, they hold promise for significantly improving clinical outcomes for patients afflicted with solid tumors.

In summary, the novel all-in-one PEG-PLL platform presented in this study [3] offers an innovative strategy for the transduction of host T cells in situ, yielding notable anti-tumor effects against solid tumors. While this breakthrough paves the way for a new paradigm in immune cell therapies, it is acknowledged that more efforts must be devoted in the forthcoming years to revolutionizing the treatment of solid tumors.

## Data Availability

Not applicable.

## Abbreviations

CAR: Chimeric antigen receptor
LVs: Lentiviruses
OVA: Ovalbumin
PEG: Polyethylene glycol
PLL: Poly-L-lysine
TCRs: T cell receptors
